# Sacro-Iliac Joint Sensory Block and Radiofrequency Ablation: Assessment of Bony Landmarks Relevant for Image-Guided Procedures

**DOI:** 10.1155/2016/1432074

**Published:** 2016-09-22

**Authors:** Trevor J. G. Robinson, Shannon L. Roberts, Robert S. Burnham, Eldon Loh, Anne M. Agur

**Affiliations:** ^1^Division of Anatomy, Department of Surgery, University of Toronto, Toronto, ON, Canada; ^2^Division of Physical Medicine and Rehabilitation, University of Alberta, Edmonton, AB, Canada; ^3^Department of Physical Medicine and Rehabilitation, University of Western Ontario, London, ON, Canada; ^4^Division of Physical Medicine and Rehabilitation, Department of Medicine, University of Toronto, Toronto, ON, Canada

## Abstract

Image-guided sensory block and radiofrequency ablation of the nerves innervating the sacro-iliac joint require readily identifiable bony landmarks for accurate needle/electrode placement. Understanding the relative locations of the transverse sacral tubercles along the lateral sacral crest is important for ultrasound guidance, as they demarcate the position of the posterior sacral network (S1–S3 ± L5/S4) innervating the posterior sacro-iliac joint. No studies were found that investigated the spatial relationships of these bony landmarks. The purpose of this study was to visualize and quantify the interrelationships of the transverse sacral tubercles and posterior sacral foramina to inform image-guided block and radiofrequency ablation of the sacro-iliac joint. The posterior and lateral surfaces of 30 dry sacra (15 M/15 F) were digitized and modeled in 3D and the distances between bony landmarks quantified. The relationships of bony landmarks (S1–S4) were not uniform. The mean intertubercular and interforaminal distances decreased from S1 to S4, whereas the distance from the lateral margin of the posterior sacral foramina to the transverse sacral tubercles increased from S1 to S3. The mean intertubercular distance from S1 to S3 was significantly (*p* < 0.05) larger in males. The interrelationships of the sacral bony landmarks should be taken into consideration when estimating the site and length of an image-guided strip lesion targeting the posterior sacral network.

## 1. Introduction

Radiofrequency ablation (RFA) of the lateral branches of the posterior sacral rami has been increasingly used to treat chronic, refractory sacroiliac joint (SIJ) complex pain [1]. Patients are selected for RFA if their SIJ pain is consistently completely or almost completely eliminated following local anesthetic block of the posterior sensory innervation. Accurate needle/electrode placement with image guidance relies on comprehensive knowledge of the course of the S1–S4 lateral branches relative to surrounding bony landmarks.

**Figure 1:**
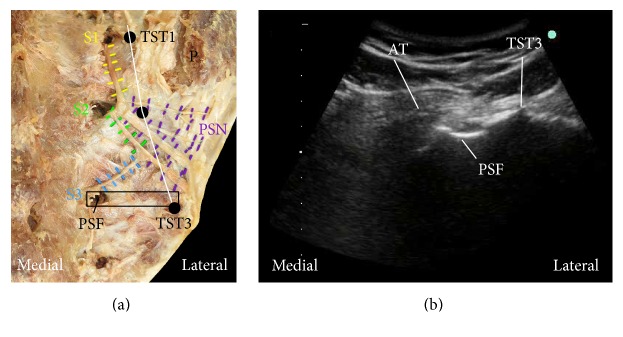
Ultrasound-guided sacro-iliac joint (SIJ) radiofrequency ablation (RFA): posterior sacral network (PSN) lateral crest technique. (a) Dissected specimen of the PSN innervating the posterior aspect of the SIJ demonstrating the location of the RFA strip lesion (white line between the first (TST1) and third (TST3) transverse sacral tubercles). The position of the ultrasound probe is indicated by the black rectangle. PSF, posterior sacral foramen; P, posterior superior iliac spine. (b) Transverse ultrasound scan showing sacral bony landmarks at the level of the third transverse sacral tubercle (TST3). Note that the increments of the scale bar on the left margin are 1 cm. AT, articular tubercle.

In a recent anatomical study related to RFA, carried out in our laboratory, the frequency and course of the lateral branches innervating the posterior aspect of the SIJ were documented using digitization and three-dimensional (3D) modeling [2]. Innervation of the posterior aspect of the SIJ was found to be from the S1–S3 ± L5/S4 lateral branches that united to form a fine nerve plexus, the posterior sacral network (PSN). The first, second, and third transverse sacral tubercles (TSTs) of the lateral sacral crest were found to be consistent bony landmarks that could be used to demarcate the superior and inferior borders of the PSN. The first to third TSTs are visible with ultrasound (US) and can be identified by using the adjacent posterior sacral foramina. Using these landmarks, a novel US-guided SIJ RFA technique was proposed that targets the PSN along the lateral sacral crest (Figure 1).

No literature was found describing the geometry and spatial relationships of the TSTs and posterior sacral foramina. Previous studies focused on quantification of the dimensions and distances between the posterior sacral foramina (Table 1). The mean heights and widths of the posterior sacral foramina and the mean interforaminal distances varied between the four studies. Two of the four studies also reported that the mean foraminal heights and interforaminal distance from S1 to S2 were larger in males than in females [3, 5].

Image-guided SIJ RFA techniques require readily identifiable bony landmarks for accurate needle/electrode placement. Understanding the relative locations of the TSTs is important when using ultrasound guidance, as the TSTs demarcate the position of the PSN innervating the posterior aspect of the SIJ [2]. However, the spatial relationships of the TSTs and posterior sacral foramina have not been quantified. Therefore, the purpose of this study was to three-dimensionally visualize and quantify the interrelationships of the TSTs, posterior sacral foramina, and auricular surfaces of the sacrum to provide an anatomical basis for image-guided SIJ block/RFA procedures.

## 2. Materials and Methods

Thirty dry adult sacra (15 M/15 F) from the teaching collection were used in this study. These sacra were selected as they were the only specimens that were completely intact without bone chipping and/or visible pathology. Ethics approval was received from the University of Toronto Health Sciences Research Ethics Board (#27210).

**Figure 2:**
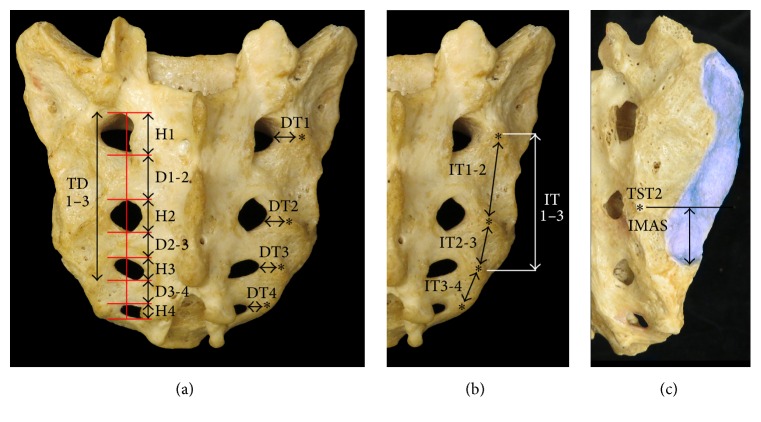
Quantification of sacral landmarks. (a) Distance from lateral margin of foramen to adjacent transverse sacral tubercle (DT), foraminal height (H), interforaminal distance (D), total distance from S1–S3 foramina (TD1–3), and transverse sacral tubercle (*∗*). Posterior view. (b) Intertubercular distance (IT) and intertubercular distance from TST1 to TST3 (IT1–3). Posterior view. (c) Distance from the level (black line) of TST2 to the inferior margin of the auricular surface of the sacro-iliac joint (IMAS). Posterolateral view.

Each sacrum was stabilized with a clamping system, leaving the entire posterior surface accessible for digitization. A MicroScribe™ G2X Digitizer (Immersion Corporation, San Jose, CA; accuracy: ±0.23 mm) was used to collect 3,000–4,300 data points per sacrum. The posterior and lateral surfaces of the sacrum, including the TSTs along the lateral sacral crest, auricular surfaces, and the circumferences of the posterior sacral foramina, were digitized. The data were imported into Autodesk® Maya® 2013 (Autodesk Inc., San Rafael, CA) and reconstructed into fully manipulable 3D models using custom plug-ins developed in our laboratory. All parameters were quantified bilaterally from the 3D models using the measurement tool in Maya®. To quantify the heights and interforaminal distances of the posterior sacral foramina, a vertical reference line through the center of each foramen, extending from the superior margin of the first posterior sacral foramen to the inferior margin of the fourth posterior sacral foramen, was used (Figure 2(a)). Each of these parameters was quantified as follows:

- Height of the first, second, third, and fourth posterior sacral foramina was measured as the distance between the superior and inferior margins of each foramen at the reference line (H1–H4, Figure 2(a)).
- Interforaminal distance was measured between adjacent posterior sacral foramina at the reference line (D1-2, D2-3, and D3-4, Figure 2(a)).
- Distance from the superior margin of the first posterior sacral foramen to the inferior margin of the third posterior sacral foramen at the reference line (TD1–3, Figure 2(a)).

The relationships of the transverse sacral tubercles to each other and to the posterior sacral foramina were quantified using the following parameters:

- Distance from the midpoint of the lateral margin of each posterior sacral foramen to the center of the ipsilateral TST of the same number (DT1, DT2, DT3 and DT4, Figure 2(a)).
- Intertubercular distance was measured between the centers of TST1 and TST2 (IT1-2), TST2 and TST3 (IT2-3), and TST3 and TST4 (IT3-4). See Figure 2(b).
- Intertubercular distance was measured between the centers of TST1 and TST3 (IT1–3, Figure 2(b)).

The relationship of the most inferior point of the auricular surface of the sacrum (i.e., the most inferior extent of the synovial part of the SIJ) to TST2 was quantified using a horizontal reference line drawn from the center of TST2 to the margin of the auricular surface. The distance between the most inferior point of the auricular surface of the sacrum and the reference line (IMAS) was measured (Figure 2(c)).

Descriptive statistics were used to summarize each parameter: (1) for all sacra and (2) by sex. The sex of each sacrum was determined using Flander's index-2 formula [7]. An independent two-tailed *t*-test (*p* < 0.05) was performed to determine if statistically significant differences were present between sexes.

## 3. Results

**Figure 3:**
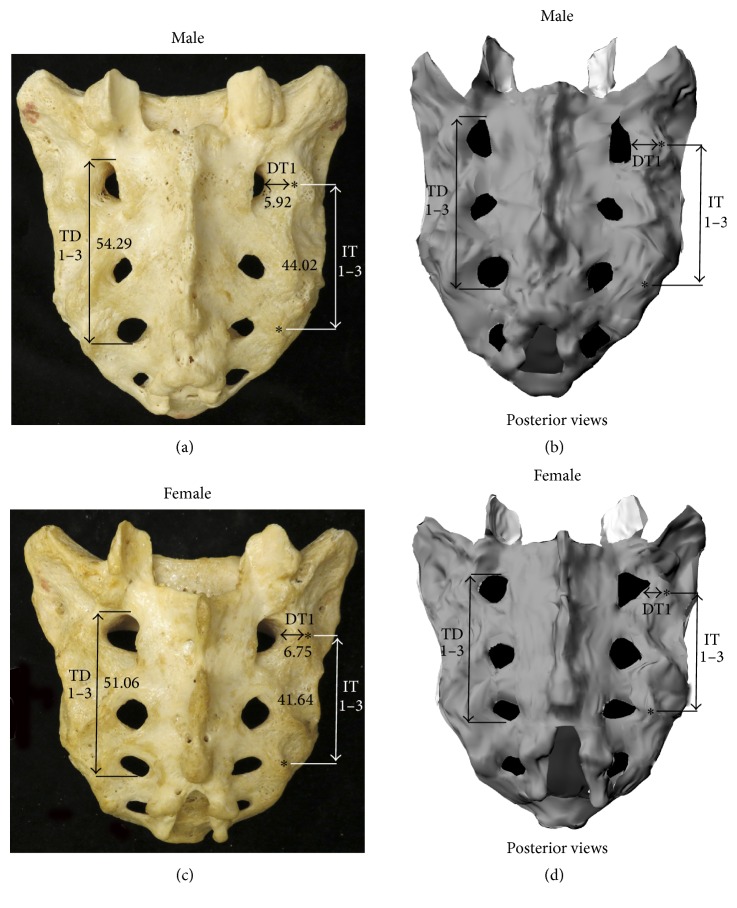
Morphology of male and female sacra. Photograph (a) and model (b) of a male sacrum; photograph (c) and model (d) of a female sacrum. DT, distance from lateral margin of foramen to adjacent transverse sacral tubercle; TD1–3, total distance from S1–S3 foramina; IT1–3, intertubercular distance from TST1 to TST3; *∗*, transverse sacral tubercle.

**Figure 4:**
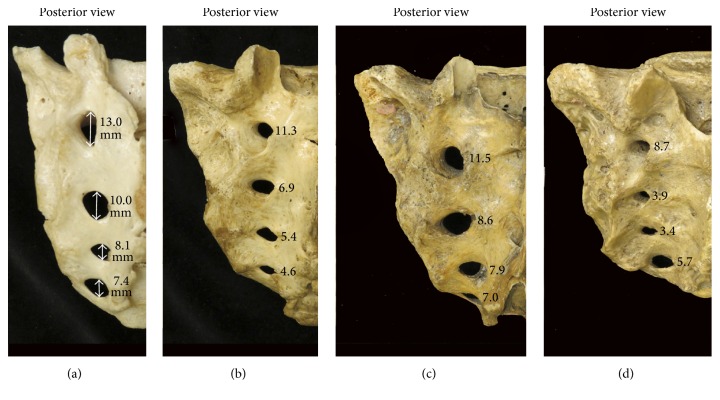
Variation in the shape and size of the posterior sacral foramina. (a) and (b) photographs of male sacra; (c) and (d) photographs of female sacra.

The shapes and sizes of the posterior sacral foramina were variable (Figures 3 and 4). In some sacra, the foramina were elongated, whereas in others they were more ellipsoidal or circular. The sizes of the posterior sacral foramina were variable, with some specimens having larger foramina and others smaller (Figure 4). Morphologically, male sacra were narrower, longer, and more triangular in shape, whereas female sacra were wider and shorter (Figure 3).

The mean heights of the posterior sacral foramina decreased in all specimens from S1 (12.22 ± 2.30 mm) to S4 (6.43 ± 1.52 mm). When considering sex differences, the mean height of each posterior sacral foramen was larger in males than in females (Table 2).

In all specimens, the mean interforaminal distance decreased from S1-S2 to S3-S4 by approximately 2 mm (Table 3). The mean S1–S3 distance measured from the superior margin of the first posterior sacral foramen to the inferior margin of the third posterior sacral foramen was 52.68 ± 5.99 mm. Both the mean S2-S3 and S1–S3 interforaminal distances were found to be significantly (*p* < 0.05) larger in males than in females.

In all sacra, the mean distance from the lateral margin of the posterior sacral foramen to the adjacent TST increased from S1 (6.34 ± 1.36 mm) to S3 (9.45 ± 2.16 mm) but decreased at S4 (7.14 ± 2.18 mm). In females, the mean distance from the lateral margin of the S1 and S2 posterior sacral foramina to the adjacent TST was larger than in males (Table 2). However, this difference was only statistically significant (*p* < 0.05) at the S1 level.

The mean intertubercular distance decreased from superior to inferior by approximately 6 mm (Table 3). The mean distance from TST2-TST3 and from TST1–TST3 was significantly (*p* < 0.05) larger in males than in females.

The inferior margin of the auricular surface of the sacrum was located inferior to TST2 (9.83 ± 3.95 mm) in all specimens except one, where it was 3.09 mm superior to TST2. There was no statistically significant difference between sexes.

## 4. Discussion

Knowledge of the relationships of the TSTs and the posterior sacral foramina provides an anatomical basis for sensory blockade of the SIJ and for estimating the length and width of strip lesions for US and fluoroscopically guided SIJ RFA procedures.

### 4.1. Bony Landmarks

In the two previous studies investigating the heights of the posterior sacral foramina, Arman et al. [4] measured the heights of the S1 and S2 posterior sacral foramina, while Ebraheim et al. [5] included S1, S2, and S3. The mean height of the S1 posterior sacral foramen found in the current study was comparable to the two previous studies (Table 1(a)). In contrast, the mean height of the S2 posterior sacral foramen was similar in the current study and Arman et al. [4], whereas that reported by Ebraheim et al. [5] was on average 4-5 mm greater at both S2 and S3 (Table 1(a)). This difference may be attributed to the radiographic measurement technique used by Ebraheim et al., resulting in possible distortion of the posterior sacral foramina on X-rays [5]. Ebraheim et al. also examined sex differences and reported that the S1, S2, and S3 posterior sacral foramina were all significantly (*p* < 0.05) larger in males than in females [5]. Similarly, in the current study, the S1–S4 posterior sacral foramina were larger in males than in females, although no statistically significant difference was found.

The interforaminal distances reported in the current study and in the literature decreased from S1 to S4 (Tables 1(a) and 3). The S1-S2 interforaminal distance was similar in all studies, but the S2-S3 interforaminal distance reported in the radiographic study by Ebraheim et al. was 5-6 mm smaller [5] than that found in the current study and in the study by Arman et al. [4]. In the current study, the S2-S3 and the total S1–S3 interforaminal distances were significantly (*p* < 0.05) larger in males than in females. This finding suggests that when using a multipolar linear strip lesion technique to ablate the S1–S3 lateral branches, a greater number of lesions may be required for male patients [8, 9].

The S2 posterior sacral foramen has been used as a reference point to demarcate the inferior limit of the SIJ for intra-articular injections [10, 11]. The results of the current study and that of Roberts et al. [2] suggest that TST2, which is clearly visible using US, could provide an alternative landmark to localize the SIJ, as the inferior margin of the joint was on average 9 mm inferior to TST2.

### 4.2. Implications for Ultrasound-Guided PSN Radiofrequency Ablation Technique

The intertubercular distance from TST1 to TST3 is clinically important as it demarcates the superior and inferior extent of the PSN innervating the posterior aspect of the SIJ [2]. Thus, the intertubercular distance from TST1 to TST3 could be used to estimate the length of an RFA strip lesion targeting the PSN. If a single multitined RF electrode was used to create a strip lesion along the lateral sacral crest, an average of four to five needle placements would be required, with a lesion diameter of 8–10 mm (Nimbus Concepts LLC). In comparison, approximately three needle placements would be required if a bipolar approach was used to generate a strip lesion between two multitined electrodes, based on the recommended interelectrode distance of 15 mm (Nimbus Concepts LLC). In the current study, the intertubercular distance from TST1 to TST3 was found to be significantly (*p* < 0.05) larger in males than in females, suggesting that males may require one additional needle placement for both approaches. The clinical significance of this finding is currently being investigated as part of a multicenter clinical study assessing the ultrasound-guided PSN lateral crest technique for sacro-iliac joint block/radiofrequency ablation.

The distance between the lateral margin of the posterior sacral foramen and the medial margin of a strip lesion along the lateral sacral crest would increase from S1 to S3 (Table 4). For example, a lesion with a diameter of 8 mm would be about 2.3 mm lateral to the posterior sacral foramen at S1, but 5.5 mm lateral at S3. If the diameter of the lesion was increased to 9 mm, the resulting lesion would be about 0.5 mm closer to each posterior sacral foramen. A medial to lateral electrode trajectory may allow the lesion to be placed slightly lateral to TST1 and further from the S1 posterior sacral foramen. This should particularly be considered in males since, at the S1 level, the distance from the posterior sacral foramen to TST1 is significantly (*p* < 0.05) smaller than in females.

## 5. Conclusions

The interrelationships of the TSTs and posterior sacral foramina were quantified to inform image-guided block and RFA of the SIJ. To ablate the PSN, the nerve plexus innervating the posterior aspect of the SIJ, the block/RFA strip lesion should extend from TST1 to TST3 (mean distance: 42.83 ± 4.72 mm). The distance from TST1 to TST3 was significantly (*p* < 0.05) larger in males than in females. The results of the current study indicate that the relationships of the TSTs and posterior sacral foramina are important considerations for estimating the site and length of a sensory block and RFA strip lesion targeting the PSN.

## Figures and Tables

**Table:** (a) Cadaveric/imaging studies

Authors	*n*	Specimens	Method	Posterior sacral foramen		Interforaminal distance (mm)
				Height (mm)	Width (mm)	
McGrath and Stringer 2011 [3]	27	Dry sacra	Photo scale bar	X	S2: 7.5 ± 1.8	S1-S2: 14.2 ± 2.7 S2-S3: 12.8 ± 1.5

Arman et al. 2009 [4]	100	Dry sacra	Vernier caliper	S1: 12.47 ± 3.16 S2: 7.62 ± 1.54	S1: 7.97 ± 1.89 S2: 7.92 ± 1.74	S1-S2: 15.92 ± 2.08

Ebraheim et al. 1998 [5]	21	X-Ray dry sacra	Ruler on X-ray	S1: 12.9 ± 0.95 S2: 12.8 ± 0.85 S3: 10.4 ± 1.55	X	S1-S2: 11.5 ± 1.35 S2-S3: 6.95 ± 0.85

Ebraheim et al. 1997 [6]	20	Cadaveric pelvis	Caliper	X	X	S1-S2: 15.7 ± 2.9

**Table:** (b) Sex comparison studies

Authors	Posterior sacral foramen				Interforaminal distance (mm)	
	Height (mm)		Width (mm)			
	Male	Female	Male	Female	Male	Female
McGrath and Stringer 2011 [3]	X	X	S2: 7.6 ± 2.0	S2: 7.3 ± 1.3	S1-S2: 14.5 ± 2.8 S2-S3: 12.7 ± 1.4	S1-S2: 13.0 ± 2.0 S2-S3: 13.4 ± 1.8

	Male^*∗*^	Female^*∗*^	Male	Female	Male	Female

Ebraheim et al. 1998 [5]	S1: 13.3 ± 0.9 S2: 13.1 ± 0.9 S3: 11.4 ± 1.4	S1: 12.5 ± 1.0 S2: 12.5 ± 0.8 S3: 9.4 ± 1.7	X	X	S1-S2: 11.9 ± 1.3 S2-S3: 7.2 ± 0.8^*∗∗*^	S1-S2: 11.1 ± 1.4 S2-S3: 6.7 ± 0.9^*∗∗*^

Significant (*p* ≤ 0.05) difference between males and females: ^*∗*^height of S1, S2, and S3 posterior sacral foramina; ^*∗∗*^interforaminal distance S2-S3. X, not measured.

**Table 2:** Quantification of parameters of posterior sacral foramina.

PSF	Mean foraminal height ± SD (mm)			Mean distance from lateral margin of PSF to TST ± SD (mm)		
	All (M + F)	Male	Female	All (M + F)	Male	Female
S1	12.22 ± 2.30	12.52 ± 2.40	11.92 ± 2.20	6.34 ± 1.36	5.92 ± 1.20^**∗**^	6.75 ± 1.41^**∗**^
S2	7.39 ± 1.61	7.70 ± 1.74	7.07 ± 1.43	7.14 ± 1.57	6.71 ± 1.56	7.21 ± 1.57
S3	6.79 ± 2.00	6.98 ± 2.10	6.59 ± 1.91	9.45 ± 2.16	9.68 ± 1.58	9.22 ± 2.62
S4	6.43 ± 1.52	6.79 ± 1.72	6.06 ± 1.22	7.14 ± 2.18	7.18 ± 2.10	7.10 ± 2.30

PSF, posterior sacral foramen (S1–S4); TST, transverse sacral tubercle; ^*∗*^significant (*p* ≤ 0.05) difference between males and females.

**Table 3:** Quantification of interforaminal and intertubercular distances.

PSF	Mean interforaminal distance ± SD (mm)			Mean intertubercular distance ± SD (mm)		
	All (M + F)	Male	Female	All (M + F)	Male	Female
S1-S2	13.58 ± 2.35	13.88 ± 2.63	13.28 ± 2.03	23.61 ± 3.46	23.96 ± 3.29	23.25 ± 3.64
S2-S3	12.71 ± 1.70	13.22 ± 1.78^*∗*^	12.19 ± 1.47^*∗*^	19.23 ± 2.68	20.06 ± 2.31^*∗*^	18.39 ± 2.80^*∗*^
S3-S4	11.25 ± 1.94	11.22 ± 2.08	11.29 ± 1.83	17.59 ± 3.03	17.84 ± 2.84	17.35 ± 3.25
S1–S3	52.68 ± 5.99	54.29 ± 6.23^*∗*^	51.06 ± 5.37^*∗*^	42.83 ± 4.72	44.02 ± 4.54^*∗*^	41.64 ± 4.67^*∗*^

PSF, posterior sacral foramen (S1–S4); ^*∗*^significant (*p* ≤ 0.05) difference between males and females.

**Table 4:** Distance between posterior sacral foramen and the medial margin of a strip lesion from TST1 to TST3.

PSF	Mean DT (mm)	Mean distance: medial margin of lesion to lateral margin of PSF (mm)								
		8 mm lesion diameter			9 mm lesion diameter			10 mm lesion diameter		
		M & F	M	F	M & F	M	F	M & F	M	F
S1	6.34	2.34	1.92	2.75	1.84	1.42	2.25	1.34	0.92	1.75
S2	7.14	3.14	2.71	3.21	2.64	2.21	2.71	2.14	1.71	2.21
S3	9.45	5.45	5.68	5.22	4.95	5.18	4.72	4.45	4.68	4.22

DT, distance from lateral margin of foramen to transverse sacral tubercle; PSF, posterior sacral foramen; TST, transverse sacral tubercle.
